# Changes in Leukogram and Erythrogram Results in Bitches with Vaginitis

**DOI:** 10.3390/ani11051403

**Published:** 2021-05-14

**Authors:** Maria Chmurska-Gąsowska, Bartosz Bojarski, Natalia Sowińska, Magdalena Strus

**Affiliations:** 1 Institute of Veterinary Sciences, University Center of Veterinary Medicine JU-UA, University of Agriculture in Krakow, 30-059 Krakow, Poland; maria.chmurska-gasowska@urk.edu.pl; 2 Institute of Ichthyobiology and Aquaculture in Gołysz, Polish Academy of Sciences, 43-520 Chybie, Poland; bbojarski@o2.pl; 3 Animal Reproduction Unit, Department of Genetics and Animal Breeding, Faculty of Veterinary Medicine and Animal Science, Poznan University of Life Sciences PULS, 60-637 Poznan, Poland; 4 Department of Microbiology, Faculty of Medicine, Medical College, Jagiellonian University, 31-121 Krakow, Poland; magdalena.strus@uj.edu.pl

**Keywords:** erythrocyte, leukocyte, blood, dog, inflammatory

## Abstract

**Simple Summary:**

Inflammatory diseases of the reproductive tract of bitches are a common problem in veterinary practice. Vaginitis, which is an inflammation of the vagina, may have various causes and degrees of severity. The aim of this study was to analyze whether the inflammation of the vagina in dogs causes changes in the parameters of white blood cells (leukogram, which is the percentage of individual types of white blood cells) and red blood cells (erythrogram, changes in the shape of erythrocytes). The obtained results suggest that leukogram and erythrogram analyses may be useful diagnostic tools in veterinary practice.

**Abstract:**

Vaginitis in female dogs is a problem most veterinarians face in their practice. It manifests as localized inflammation, and its variable etiology and different severities often make diagnosis problematic. The study consisted of comparing blood smears taken from 16 animals: 8 healthy bitches and 8 bitches with confirmed vaginitis. We analyzed the percentage of different types of white blood cells (leukogram) and changes in the shape of red blood cells (erythrogram) in both groups. We observed changes in red blood cell morphology, i.e., a higher percentage of lacrimocytes and schistocytes in female dogs with vaginitis compared to their healthy counterparts. The observed hematological changes may illustrate the severity of inflammation. The analysis of erythrograms showed a significantly higher percentage of lacrimocytes and schistocytes in diseased bitches (1.58 ± 1.19% and 0.13 ± 0.12%) compared to healthy animals (0.58 ± 0.38 and 0.00 ± 0.00, respectively). The obtained results may indicate that the analysis of erythrograms throughout the course of vaginitis in bitches may constitute a diagnostic tool, as opposed to the analysis of leukograms, which is more sensitive when it comes to the systemic inflammatory response of the organism. It seems that simultaneous analysis of erythrograms and leukograms may facilitate the diagnostic process in clinical practice.

## 1. Introduction

Vaginitis is a disease that does not occur frequently, although any practicing veterinarian is likely to encounter it. Vaginitis can occur in any age or breed, and in both intact and spayed bitches. The causes of vaginitis can be very diverse, and include bacterial infection, viral infection (e.g., herpes virus type I–HVC I), fungal infection (however, it is very rare), hyper- or hypoestrogenism, urinary tract infection or urinary incontinence, genital infections such as pyometra, metritis or uterine stump abscess, vaginal trauma, chemical irritation due to urovagina, mechanical irritation caused by foreign bodies or tumors, anatomic abnormalities of the genitourinary system and vaginal atrophy after being spayed [1,2]. We distinguish juvenile vaginitis from adult bitch vaginitis depending on age and sexual maturity. Juvenile vaginitis comprises 40–52% of reported cases of vaginitis. Affected animals usually do not demonstrate systemic involvement, and immaturity of the reproductive and immune system are considered to be its causes [3]. Bacterial infection, which is the most common case of adult bitch vaginitis, is usually caused by the overgrowth of the normal microbiota of the vagina [2]. In our previous study, we reported that *Escherichia coli*, *Staphylococcus pseudintermedius*, *Streptococcus canis*, *Enterococcus* spp., and *Mycoplasma* spp. are the most commonly isolated bacteria from both healthy and infected (vaginitis) bitches [2]. Other authors have also shown the presence of *Proteus mirabilis* and *P. vulgaris*, *Pseudomonas aeruginosa*, *Haemophilus,* and *Pasteurella haemolytica* [1].

Vaginitis may be self-limiting, and treatment, when necessary, includes therapy with an antibiotic (or antibiotics), vaginal cleaning, and/or surgical correction of predisposing abnormalities. If left untreated or if improperly treated, it may lead to subfertility or infertility [1].

The analysis of hematological parameters is one of the basic elements in assessing the state of health in both human [4] and veterinary medicine [5]. Changes in hematological parameters can be physiological, may be a result of environmental stress factors, or may accompany various diseases [6,7,8,9,10,11,12,13]. Uyarlar et al. [14] showed that dairy cows with ketosis exhibited a higher number of total leukocytes, neutrophils, and monocytes in comparison to control individuals. Lambert et al. [12] revealed a connection between inflammatory response syndrome in horses and the presence of a toxic neutrophil, both of which were more common in diseased animals than in healthy individuals. In addition, leukocytosis, neutrophilia, and lymphopenia were reported in dogs with pyometra [15]. Stacy et al. [16] revealed that the inflammatory process may result in morphological changes in white blood cells in Asian and African elephants (*Elephas maximus* and *Loxodonta africana*). Silva et al. [17] showed the occurrence of erythrocyte deformations (cell membrane scrambling, cell shrinkage, and membrane blebbing) in Swiss mice infected with *Salmonella enterica* serovar Typhimurium. Christopher et al. [11] found higher percentages of schistocytes, microcytes, keratocytes, and spherocytes in rabbits with different organ and systemic diseases in comparison to healthy individuals. It is known that metabolic syndrome induces changes in the shape of red blood cells in humans [18].

To our knowledge, the data regarding the relationship between inflammation in dogs and hematological changes are insufficient. The effect of vaginitis on hematological indices, especially on the morphology of erythrocytes, is still unknown. Thus, the aim of the current study was to investigate if vaginitis results in changes in leukogram and/or erythrogram (erythrocyte morphology) outcomes in bitches.

## 2. Materials and Methods

### 2.1. Animals

Client-owned dogs were transferred to the Veterinary Clinic of the University Center of Veterinary Medicine JU-AU, University of Agriculture in Cracow, Poland in connection with reproductive problems, estrous monitoring, determination of mating date, or routine gynecological examination of breeding bitches. Eight healthy bitches (4 in proestrus/oestrus, 1 in diestrus and 3 in anestrus) and eight bitches with vaginitis (2 in proestrus and 6 in anestrus) were qualified for this study (mixed breeds, aged from 1 to 7 years). The qualification standards were set according to a protocol described by Golińska et al. [2]. Briefly, qualification was based on a clinical and gynecological examination, which included vaginal cytology. Subsequently, the phase of the estrous cycle was determined according to the guidelines described by Concannon [19]. These examinations were carried out by an experienced veterinarian. The bitches with mucusy, milky-white, yellowish, or greenish vaginal discharge and the presence of numerous neutrophils in a cytological smear were classified as animals with vaginitis. In bitches with vaginitis, owners often observed increased licking of the vulva by the animal and polydipsia/polyuria. Moreover, strands of mucus in the cytological smear were visible. The bitches without vaginitis had physiological vaginal discharge (from blood to straw colored) or no discharge at all. The presence of a few neutrophils in the cytological smear was acceptable in two cases: at the beginning of the proestrus phase (during bleeding) and during the diestrus phase, when the bleeding had ceased. To the best of our knowledge, no additional detailed classifications of canine vaginitis have been published to date. Only bitches in good condition, showing no systemic or organ diseases (excluding symptoms of vaginitis), were selected for this study, and bitches undergoing any treatment were excluded. The animals were not given antibiotics for at least 2 weeks prior to collection. The owners were informed about the purpose of the study and gave their written consent for their dogs to participate in the study.

### 2.2. Sample Collection

**Figure 1:**
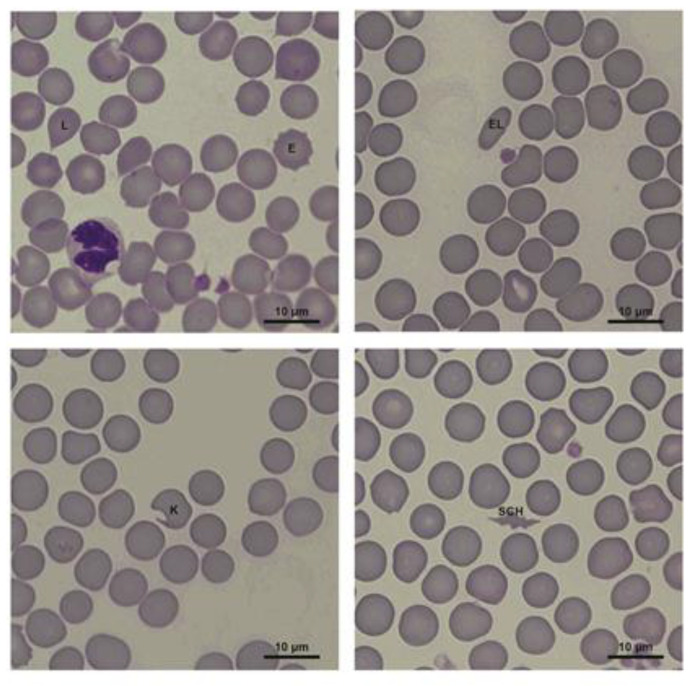
Erythrocyte morphology changes in bitches (E—echinocyte, EL—elliptocyte, K—keratocyte, L—lacrimocyte, SCH—schistocyte).

The blood for hematological tests was collected from 8 healthy (control) and 8 diseased bitches from the cephalic vein into EDTA plastic tubes. Analyses were performed using the LaserCyte, Vetlab station (IDEXX, Westbrook, ME, USA) with settings for canine blood after testing for trueness and precision. Next, blood smears were prepared. The smears were stained with a modified Wright Giemsa stain (Hemacolor^®^ staining kit, Merck, Darmstadt, Germany) according to the manufacturer’s instructions. After drying, the smears were analyzed under optical microscopes, Leica DM2500 (Leica Microsystems Inc., Buffalo Grove, IL, USA) and Nikon Eclipse Ci (Nikon Instruments Inc., Melville, NY, USA), for leukogram and erythrogram determination, based on the hematological atlas [20]. The leukogram included differentiation of individual types of leukocytes into: segmented neutrophils, band neutrophils, hypersegmented neutrophils, toxic neutrophils, non-activated lymphocytes, activated lymphocytes, monocytes, eosinophils, and basophils; 100 white blood cells were analyzed each time. The differentiation of erythrocytes into normal (unchanged) and altered (with a changed shape) cells was included in the erythrogram. The following cell types were classified as altered erythrocytes: acanthocytes, echinocytes, elliptocytes, keratocytes, lacrimocytes, schistocytes, and spherocytes, as well as poikilocytes (erythrocytes of irregular shapes that could not be classified into any of the above categories). Each time, 600 red blood cells were analyzed. The altered erythrocytes are shown in Figure 1.

### 2.3. Statistical Analysis

Due to the lack of compliance of the analyzed data with the normal distribution, the data were analyzed statistically using a non-parametric Mann–Whitney U test. The level of significance was set at α = 0.05. The analysis was conducted with the Statistica software. The results of the analyses are presented as percentage values (mean ± SD).

## 3. Results

The analysis of leukograms did not reveal statistically significant differences. We failed to detect basophils in any of the studied animals (only in one diseased individual was 1 basophil observed in 100 analyzed cells) (Table 1).

The analysis of erythrograms showed a significantly higher percentage of lacrimocytes and schistocytes in diseased bitches (1.58 ± 1.19% and 0.13 ± 0.12%) compared to healthy animals (0.58 ± 0.38 and 0.00 ± 0.00, respectively). No acanthocytes or spherocytes were observed in either of the animals (Table 2).

## 4. Discussion

Our study did not reveal any significant effects of vaginitis on the leukogram in the case of female dogs. It is known that the phase of the estrus cycle has no influence on hematological parameters [21]. Bauer et al. [22] noticed that carrageenan-induced local aseptic moderate inflammation did not lead to a significant systemic inflammatory response in bitches. Despite the fact that our study involved infectious inflammation as opposed to the aseptic inflammation studied by Bauer et al. [22], and the authors themselves strongly emphasized that their empirical study involved only five dogs, the results were comparable. The increase in toxic neutrophil counts in horses with systemic inflammatory response syndrome (SIRS) observed by Lambert et al. [12] may be a result of the release of band cells from the bone marrow, which can occur in acute systemic inflammatory processes [23]. According to Aroch et al. [24], analysis of neutrophil cytoplasmic toxicity can provide useful clinical information and may serve as a good prognostic predictor in dogs with different pathological states. Saleh and Allam [25] observed that Barki ewes diagnosed with pneumonia exhibited higher counts of neutrophils, eosinophils, basophils, and monocytes in comparison with healthy individuals. It was observed that leukocytosis with left shift was more marked in dogs with closed cervix pyometra complex than in dogs with open cervix pyometra complex (with discharge of pus) [15,26].

Our study showed that localized inflammation contributed to a significantly increased percentage of lacrimocytes and schistocytes in the erythrogram. Although many authors have indicated changes in the basic hematological parameters, such as RBC count, Hb concentration, and Ht values in the course of different inflammatory diseases in animals [15,17,25], studies concerning changes in the erythrogram are very scarce. Acanthocytosis is often seen in cats with liver disorders [27]. Acanthocytes were also detected in dogs with hemangiosarcoma, glomerulonephritis, as well as disseminated intravascular coagulation [28]. Leptocytes and codocytes were noted in hepatic insufficiency, especially in dogs and cats with portocaval shunts [27,29]. Sepsis leads to decreased red blood cell deformability in rats and mice [30,31] and altered erythrocyte morphology, with the formation of echinocytes, sphero-echinocytes, and spherostomatocytes [32]. These changes are thought to either be a combined result of oxygen free radicals and toxin action, or depletion of ATP in cells [32]. It is also known that the erythrocyte membrane interacts with some inflammatory molecules, which may result in eryptosis phenomena such as cell shrinkage, membrane deformities, and other structural disorders [17,33].

## 5. Conclusions

Changes in both the leukogram (percentages of individual types of white cells) and the erythrogram (changes in the shape of erythrocytes) seem to constitute significant markers of homeostasis disruptions in mammals. The analysis of our results and the results obtained by other authors may suggest that leukograms are sensitive markers of systemic inflammatory response, while erythrograms seem to change both in the localized and systemic inflammatory process. Neither changes in the leukogram nor changes in the morphology of erythrocytes are specific. Thus, further studies are necessary to assess the clinical utility of these markers. The presented studies are preliminary, and we are aware that they are only an introduction to the development of a diagnostic method. To the best of our knowledge, no studies on the relationship between vaginitis and the erythrogram and leukogram have been published so far.

## Figures and Tables

**Table 1:** Leukogram results of healthy and diseased bitches.

Types of Leukocytes [%]	Healthy Individuals (mean ± SD)	Diseased Individuals (mean ± SD)	*p*-Value
segmented neutrophils	54.00 ± 9.70	46.88 ± 9.51	0.234
band neutrophils	4.00 ± 3.85	5.75 ± 2.96	0.328
hypersegmented neutrophils	2.75 ± 2.19	2.50 ± 4.84	0.195
toxic neutrophils	5.38 ± 6.02	7.50 ± 5.90	0.505
non-activated lymphocytes	24.50 ± 16.25	26.25 ± 6.65	0.505
activated lymphocytes	2.00 ± 1.60	2.25 ± 1.98	0.878
monocytes	7.25 ± 4.06	8.25 ± 2.49	0.645
eosinophils	0.13 ± 0.35	0.50 ± 1.07	0.645
basophils	0.00 ± 0.00	0.13 ± 0.35	0.721

**Table 2:** Erythrogram results of healthy and diseased bitches.

Types of Erythrocytes [%]	Healthy Individuals (mean ± SD)	Diseased Individuals (mean ± SD)	*p*-Value
normal erythrocytes	97.17 ± 1.79	92.40 ± 6.15	0.065
acanthocytes	0.00 ± 0.00	0.00 ± 0.00	not applicable
echinocytes	1.73 ± 1.65	5.08 ± 5.91	0.505
elliptocytes	0.31 ± 0.33	0.63 ± 0.52	0.195
keratocytes	0.06 ± 0.12	0.04 ± 0.08	0.959
lacrimocytes	0.58 ± 0.38 ^A^	1.58 ± 1.19 ^B^	0.038
schistocytes	0.00 ± 0.00 ^A^	0.13 ± 0.12 ^B^	0.038
spherocytes	0.00 ± 0.00	0.00 ± 0.00	not applicable
irregular erythrocytes (poikilocytes)	0.15 ± 0.14	0.15 ± 0.14	1.000
all changed	2.83 ± 1.79	7.60 ± 6.15	0.065

Values in rows marked with different letters (A or B) differ significantly (*p* < 0.05).

## Data Availability

The data presented in this study are available in this paper.
